# Risks of Drone Use in Light of Literature Studies

**DOI:** 10.3390/s24041205

**Published:** 2024-02-13

**Authors:** Agnieszka A. Tubis, Honorata Poturaj, Klaudia Dereń, Arkadiusz Żurek

**Affiliations:** 1 Department of Technical Systems Operation and Maintenance, Faculty of Mechanical Engineering, Wroclaw University of Science and Technology, Wyspianskiego Street 27, 50-370 Wroclaw, Poland; honorata.poturaj@pwr.edu.pl; 2 Unmanned Aerial Vehicles (UAV) Section, Center for Advanced Systems Understanding Autonomous Systems Division, Helmholtz-Zentrum Dresden-Rossendorf e.V. (HZDR), Untermarkt 20, D-02826 Görlitz, Germany; k.deren@hzdr.de (K.D.); a.zurek@hzdr.de (A.Ż.)

**Keywords:** literature review, PRISMA, UAV, unmanned aerial vehicles, risk assessment

## Abstract

This article aims to present the results of a bibliometric analysis of relevant literature and discuss the main research streams related to the topic of risks in drone applications. The methodology of the conducted research consisted of five procedural steps, including the planning of the research, conducting a systematic review of the literature, proposing a classification framework corresponding to contemporary research trends related to the risk of drone applications, and compiling the characteristics of the publications assigned to each of the highlighted thematic groups. This systematic literature review used the PRISMA method. A total of 257 documents comprising articles and conference proceedings were analysed. On this basis, eight thematic categories related to the use of drones and the risks associated with their operation were distinguished. Due to the high content within two of these categories, a further division into subcategories was proposed to illustrate the research topics better. The conducted investigation made it possible to identify the current research trends related to the risk of drone use and pointed out the existing research gaps, both in the area of risk assessment methodology and in its application areas. The results obtained from the analysis can provide interesting material for both industry and academia.

## 1. Introduction

Drones have been used for many years in various military missions [1]. However, advancing technological developments, including those associated with Industry 4.0, have led to the increased use of unmanned aerial vehicles (UAVs, also known as drones) in non-military areas. Among these areas, logistics, public safety, traffic surveillance, and monitoring are the most commonly cited [2]. As Yoo et al. note [3], the popularity of drone use in logistics areas has been mainly influenced by their use by large online retailers (Amazon, Google, DHL, Walmart) in the parcel delivery process. These researchers also note that drone delivery’s main benefits are its speed, cost-effectiveness, and environmental friendliness [3].

The application area of drones is one of the criteria for their classification in literature reviews. An example of this approach is the classification presented by Singhal et al. [4]. The authors of this classification have defined three primary groups of drones, the membership of which being determined by the application area of the device. Thus, a distinction is made among the following [4]:

- Civilian group—refers to civilian applications of drones and includes drones used in such areas as photography, construction, mining, delivery, agriculture, logistics, disaster management, and surveillance.
- Environment group—concerned with the use of drones for ecosystem monitoring. This includes drones used primarily in the areas of soil monitoring, crop monitoring, water, underwater, mountain inspection, and air quality monitoring.
- Defence group—concerned with the use of UAVs in military applications. This includes drones used in the area of combat aircraft, spying, bomb dropping, missile launching, surveillance at the border, and warzone medical supplies.

The presented classification also shows the intensive development of drone use in non-military areas. Another widespread criterion for classifying drones is size [5].

Confirmation of the increasing importance of drone use in non-military areas is the growing number of publications on drone-related literature reviews. For this article, a search was conducted in the Web of Science database according to the following query:

TITLE_ABS_KEY (drone) AND (review).

**Figure 1:**
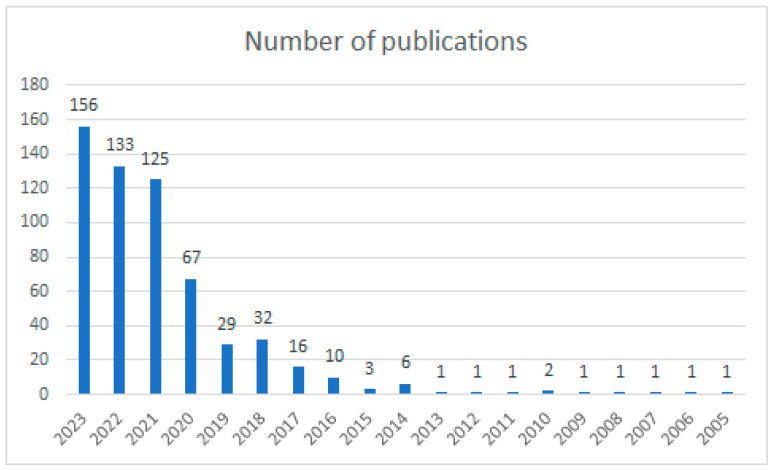
Number of publications of review articles on the use of drones according to Web of Science.

This proceeding identified as many as 588 papers meeting the accepted search criterion. As shown in Figure 1, an increase in the number of such literature reviews has already been recorded since 2016, and as of 2021, the number of published review articles is more than 100 documents per year. This confirms the growing importance of such reviews in literature studies on using drones in various systems.

Published literature reviews cover various issues related to using and applying UAV systems. However, there are two leading research trends regarding literature studies. Both of these trends are presented in Table 1, along with examples illustrating the scope of the literature reviews performed.

The authors’ analysis of review articles shows that, in the last five years, literature studies on the risks of drone use have been published only regarding their application in a specific area, e.g., [23,35]. However, the purpose of these publications was not to cover topics related to methods and techniques for assessing the risks of drone use, but only to analyse the opportunities and risks of drone application in the selected area. In contrast, the purpose of our research is to develop a framework for a method of assessing and managing the risks associated with drone operations. Therefore, we analysed 256 documents we obtained while searching the Web of Science database for publications related to the keywords “drone” + “risk”. This article aims to present the results of the bibliometric analysis of these documents and discuss the main research streams related to the topic of risk in drone applications. The main contributions of this article include the following:

- The results of the bibliometric analysis show contemporary publication trends related to the topic of risk in the application of drones.
- The proposed thematic classification for research areas related to drone application risk.
- Arrangement of publications related to drone application risks according to the proposed research classification.
- Identification of current research gaps in publications related to drone application risks.

**Figure 2:**
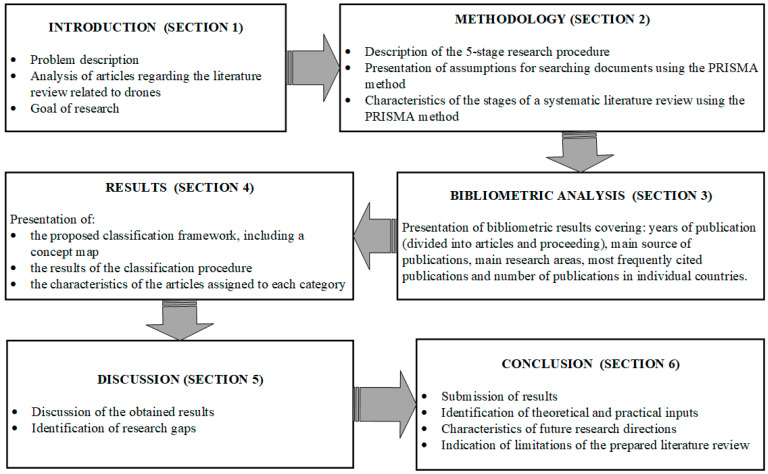
Article structure.

The structure of this article is shown graphically in Figure 2.

## 2. Methodology

### 2.1. Stages of the Research Procedure

**Figure 3:**
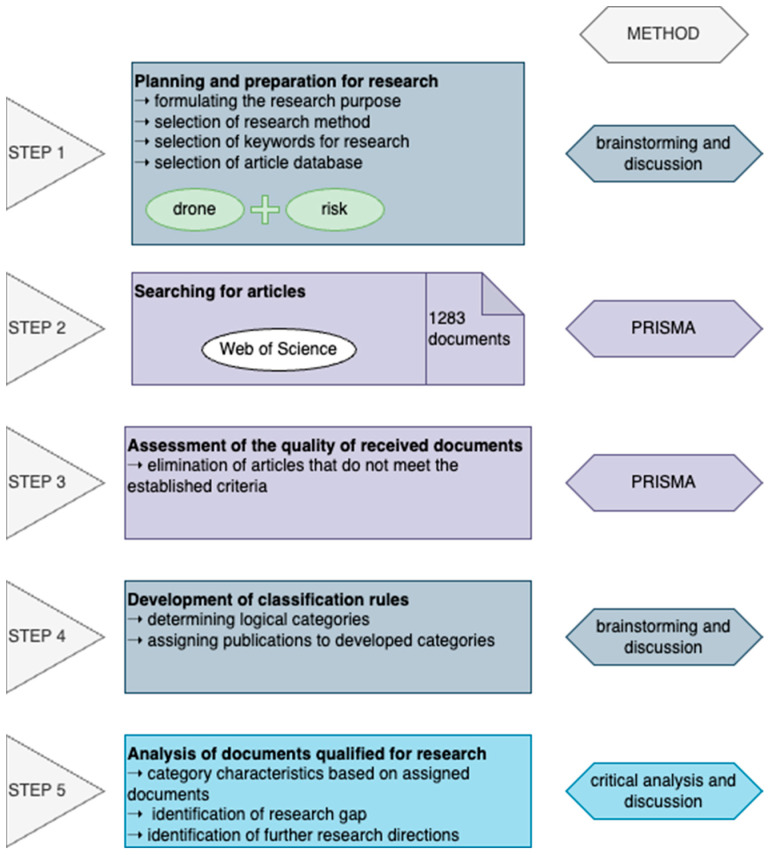
Methodology of research.

Preparing a reliable literature review requires appropriate substantive preparation and adapting appropriate research methods to the planned work. This study was divided into five stages, according to the graphics shown in Figure 3. 

In the first stage, a discussion was held, which was preceded by a brainstorming session, allowing us to formulate the purpose of this study. Then, the method necessary to search and catalogue the documents was selected. The most extended discussion concerned the selection of keywords. The word “risk” was beyond doubt. However, the selection of the appropriate word to describe flying vehicles required a trial search of the database. A search using the abbreviation “UAV” returned fewer than 130 documents. The search using the keyword “drone” yielded over 1000 records, including all the documents from the first search. The database to be searched was the Web of Science Core Collection (WoS), which received a larger number of articles than the Scopus database.

In the second and third stages, the PRISMA (Preferred Reporting Items for Systematic Reviews and Meta-Analyses) method was used to conduct a systematic review in accordance with the guidelines described by Moher et al. [36]. A detailed description of the procedure can be found in Section 2.2.

After selecting the documents, a brainstorming session was organised to determine logical categories. The following discussion allowed for the selection of seven categories: cybersecurity, drones as a source of risk, technology development related to the operation of drones, preventive activities against the risks associated with drones, monitoring, other applications of drones, and opinion surveys. Articles that did not fit into any of these main categories and were unique in their research area were assigned to the other category.

The documents qualified for review in stage three and assigned to categories were analysed in detail. This made it possible to confirm the articles belonging to categories and create descriptions based on them. In addition, a research gap was identified, determining future research directions on UAV topics.

### 2.2. PRISMA

**Figure 4:**
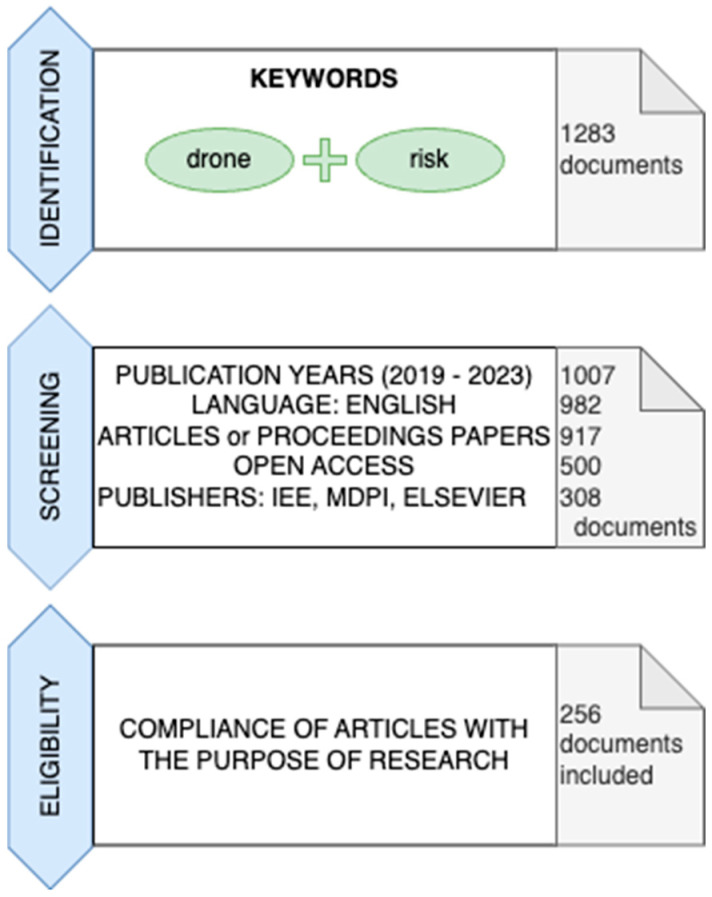
PRISMA—diagram of the systematic selection of literature.

The use of the PRISMA methodology requires the search to be carried out following the authors’ recommendations. The search involves a four-phase flow (identification, screening, eligibility, and inclusion), and its results are schematically illustrated in Figure 4.

As part of the identification, 1283 records were received from the WoS database. There were no duplicates in the search. All records were screened. The first limitation was the year of publication; documents published from 2019 to 23 November 2023 (the search date) were selected. Only documents published in English were selected. It is a language used by scientists worldwide, allowing them to share knowledge and discoveries. This study focuses on articles and proceedings papers available under open access. Both the choice of language and open access increase the availability of the results and enable people interested in the topic to access the source materials. At the very end of the screening process, articles published by three leading publishers in this area were selected: IEEE (Institute of Electrical and Electronics Engineers), MDPI, and Elsevier. The selection of the publishers mentioned above also guarantees the publications’ high quality and relevance. Ultimately, 308 articles advanced to the next PRISMA stage.

Subsequently, it was checked whether the selected publications were compatible with the purpose of this study, specified at the beginning. In some articles, the drone appeared as an example of innovative technology that could be used in the described study, but no further description of this application was provided. UAVs often appeared as one of the tools used in Industry 4.0, most often in the abstract and in the introduction to an article whose main content was about topics unrelated to drones. A total of 256 documents were qualified for this study.

## 3. Bibliometric Analysis

**Figure 5:**
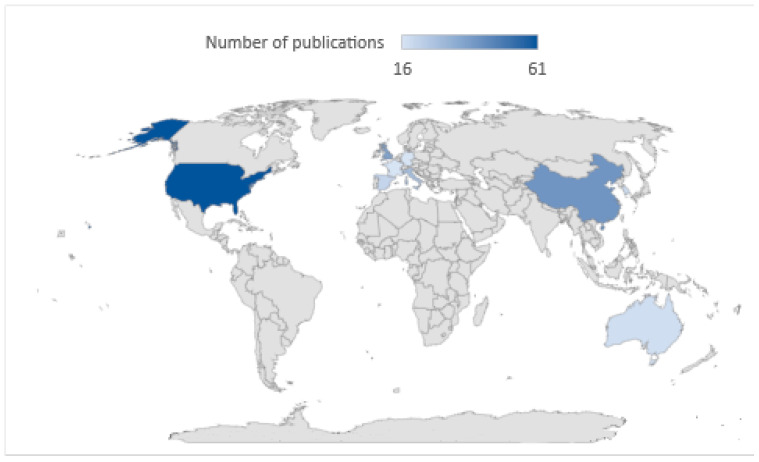
Countries leading in drone publishing.

Scientists from research centres around the world publish papers discussing the design and use of drones. However, there are leading countries in this field, such as the United States, China, and Great Britain. The map in Figure 5 shows the countries where the most publications on the researched topic occurred.

**Figure 6:**
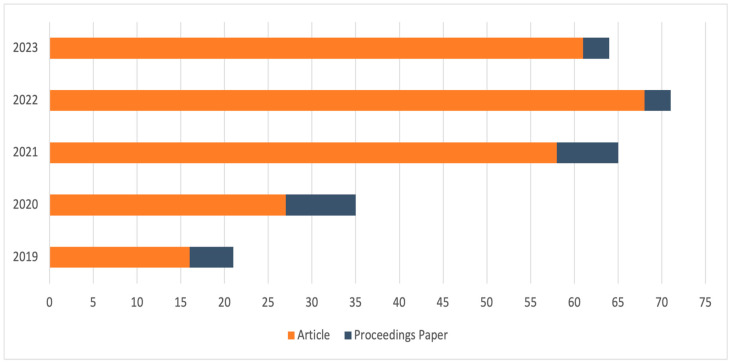
Years of publication and types of documents.

The bibliometric analysis concerns 256 articles selected for this study. This allows the presentation of selected documents in a quantitative manner. Following the limitation that specifies the year of publication for the article, the number of published articles and conference materials distributed over the selected years was checked. The graph in Figure 6 shows that after 2020, there was a significant increase in publications regarding drones. This increase is due to the development of technology; the search was performed on 23 November 2023, so 2023 should culminate with several publications similar to those in 2022. Interestingly, during the COVID-19 crisis, drone potential was further harnessed, using the people-free nature of the technology to modify current service delivery to improve safety and capacity levels. This included the delivery of face masks to remote islands in Korea and prescription medicines from pharmacies to retirement villages in Florida. It could be argued that COVID-19 has increased technological advancement in many areas, and that perhaps drones represent a revolution in how we transport goods and potentially even ourselves (however, such an analysis is reserved for a future paper) [37]. Most of the documents on the analysed subject were published in the form of articles; proceedings papers constitute only 10%. This shows that drones are a widely researched topic but are not presented at conferences; it is possible that there are no conferences where drones are included as part of the subject.

The leading magazine publishing on drones was “Drones” by MDPI publishing house: 20% of publications. It is an international magazine focusing on the design and application of UAVs and the construction of their systems. The next five leading magazines on this topic are presented in Table 2. Unsurprisingly, these magazines focus on modern technologies and their application in a sustainable environment.

As part of the bibliographic analysis, the research areas to which publications about drones were assigned were checked. Table 3 shows a summary of this analysis. Some publications were assigned to two or more areas, so it is impossible to provide the percentage share of individual areas in the total number of documents analysed. Most publications are assigned to “engineering”, which is an obvious choice when analysing mechanical devices such as UAVs. The following areas concern research related to drone software, such as “remote sensing” and “computer science”. It is important that the most frequently selected areas include “Environmental Sciences & Ecology”. This shows how important it is to adapt drones to sustainable and ecological technology development principles.

UAVs are a current topic, which is confirmed by numerous citations of selected items. Table 4 shows the most frequently cited publications. The articles with the highest number of citations were published in 2020 or 2021. The large number of citations accumulated over two or three years shows that these drone applications have numerous applications in both practice and research.

Publications about drones appear worldwide; their number and citations clearly show that this is a promising area of research.

## 4. Results

**Figure 7:**
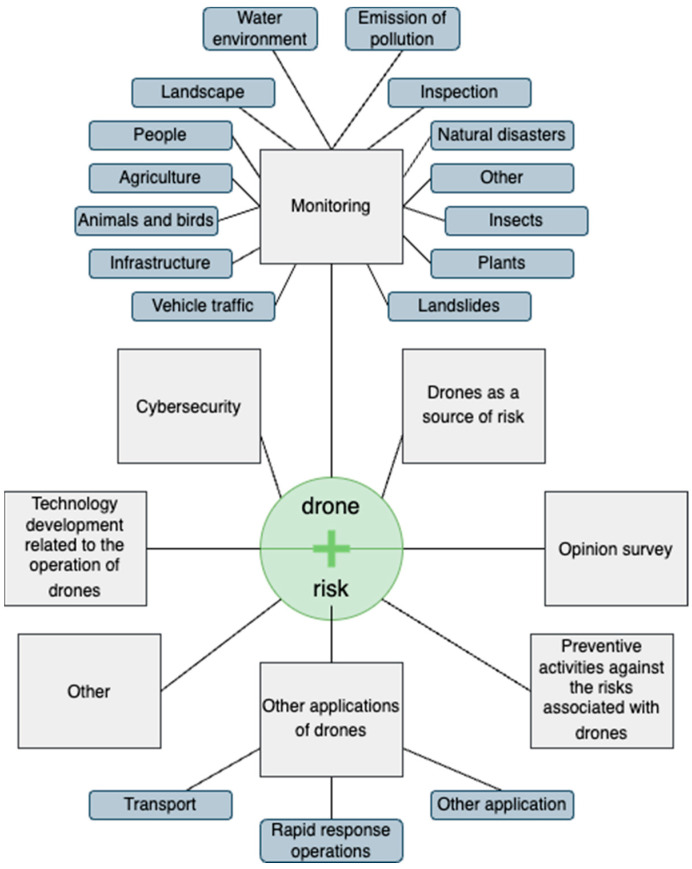
Map of the research categories.

The preliminary analysis identified eight basic categories (with two categories also distinguished by subcategories) that illustrate current research trends on drone risks. The proposed thematic groups are shown in Figure 7, while Table 5 presents the results of the classification procedure carried out for the analysed set of documents.

Each research category is characterised below.

### 4.1. Monitoring—102 Documents

The most popular topic in the analysed set of publications is research on the use of drones in monitoring specific phenomena. As many as 102 documents were classified in this group. The research characteristics, as described in these publications, are presented in Table 6 due to the size of this group. It is also worth noting that a detailed analysis of publications in this area made it possible to distinguish research subcategories for this set.

The analysis of the above publications made it possible to conclude that in the process of monitoring, the use of drones serves as a supplement to the measurement for the field surveys carried out (the field surveys are carried out first and then supplemented with data from the drone) or as a system reporting the need for additional research (identifying a specific phenomenon that requires additional research). The authors of the publication point out in their research the benefits of drone use, such as:

- The ability to conduct territorial surveys over a larger area in a shorter time.
- Lower costs of conducted surveys in the field.
- Increased safety of performed surveys in dangerous and difficult-to-access areas.
- The ability to obtain real-time images and immediately analyse them for decision-making.

It is also worth noting that drones are often used only as tools to take pictures/measurements. Then, the data they collect are analysed based on machine learning algorithms or artificial intelligence. Therefore, many publications in this group refer to combined autonomous data collection and analysis systems based on drones and AI. Of particular relevance is the use of such solutions for monitoring natural disasters in progress, such as fire [51,52,53] and flooding [43,52,53,54,55,56], in which the decisions made can save lives. The second combination often found in publications about drones used for monitoring is the combination of drones with laser scanner technology (LiDAR) for surveying. An analysis of the publications shows that maps created from data collected by drones are based either on images taken with cameras of various types or on scans derived from LiDAR.

Drone monitoring is also used very often to conduct inspections. Due to the efficiency of performing tasks with reduced turnaround time, the ability to add to places that are difficult for humans to access, and the lack of contraindications to work in harsh conditions, drones are increasingly being used as a tool for performing various types of inspections [126]. Examples of such publications relating directly to inspection processes based on drone-based monitoring are as follows:

- Conducting inspections of unburied land pipelines [127];
- Identifying corrosion in industrial structures such as telecommunications towers [128] and wind farms [129,130];
- Conducting inspections of safety-critical infrastructure [131];
- Tracking construction progress [132,133];
- Inspecting terrain for unexploded ordnance [134] and landmines [135];
- Inspecting bridge and road infrastructure [38,136].

### 4.2. Other Applications of Drones—48 Documents

Monitoring is the main area of non-military application of drones. However, researchers and industry are constantly looking for new areas where the potential of autonomous aerial systems can be exploited. Drones are increasingly used in rapid response operations, where response time is critical to determining success. As indicated in the introduction, more and more logistics operators and online retailers are interested in using drones to deliver business and individual shipments to consumers [3].

#### 4.2.1. Rapid Response Operations

Construction, ease of implementation, efficiency, safety, effectiveness, and the ability to transport items or collect data cause drones to be considered a promising technology for search and rescue missions [143,144,145,146,147,148,149,150], support for ground transport in dire situations [151,152,153], or disaster respond and recovery [143,144,146,154,155,156]. Drones can be used for the following purposes:

- Detection of chemical substances [155];
- Disaster victim identification [150];
- Firefighting operations [157,158];
- Transport of medical products [151,159,160];
- Restoring radio communication [149];
- Support for police patrols [161];
- Responding to the gestures of a person participating in a response mission [162];
- Surveillance in areas with diverse radiation levels [163];
- Detecting anomalies in pollution [113].

Drones have been adapted to the needs created by the COVID-19 pandemic [39], which shows the multitude of possible applications and the flexibility in adapting drones to the emerging or changing needs of the user. During COVID-19, drones were used, among others, for transporting medical supplies, facilitating contactless deliveries, or disinfecting large surfaces [164]. Another example is the system described in [151], which could be refined and used for monitoring elderly patients and delivering medical supplies to them as soon as possible.

#### 4.2.2. Transport

The use of UAVs to transport materials is marked by advantages such as reduced costs, increased safety, and increased efficiency [166,174,176]; however, not everything can be transported this way. Researchers are exploring the possibilities of using UAVs for transportation while noting their limitations. Drones make it possible to transport goods from point A to point B in a delivery service [165,166,167,168]. It is also possible to transport medical goods or blood, in compliance with existing regulations [152,153,160,169,170,171,172]. There are many opportunities for commercial use of drones; an overview using Australia as an example can be found in [173]. Using drones to transport and spread materials is also possible in other fields, such as in agriculture [174] or construction [175].

#### 4.2.3. Other Applications

As noted in the introduction, drones are currently used for military [177,178] and non-military missions. The scope of their use is increasing every year, so there are publications in the literature on newer and newer drone applications in areas such as:

- Warehouse operations support [179,180];
- Education [181];
- Mobile communications [42,182].

Some studies on the use of drones describe very interesting, although infrequent, situations such as:

- Fighting mosquitoes [183] or desert locusts [184];
- Exploration of other planets, such as Mars [185];
- Rehabilitation of birds of prey [186];
- Herding wild horses into pens [187].

### 4.3. Technology Development Related to the Operation of Drones—43 Documents

Research on UAVs concerns possible applications and the technology necessary for their proper functioning. An overview of key technologies enabling the development of systems using drones is described in [203]. While [152] describes the technological or regulatory challenges for UAVs, in [204,222], attention is drawn to the legal-political, socio-legal approach and the implementation of regulations, highlighting the measures that should be taken before making a given technology available to the public. Some of the important areas are planning paths, avoiding obstacles, and navigation [143,154,156,188,189,190,191,192,193,194,195,196,197,198,199,200,201,202]. The aspects mentioned above have a significant impact on the operational safety of the drone. The increase in safety may also result from changes in the design [205,206], appropriate risk analysis tailored to the purpose of the drone [163,188,189,202,207,208], and the detection of unexpected behaviours and abnormal situations during the operation of the drone [209,210,211,212,213]. Just as important as the drone’s flight is its landing. Researchers pay attention to automatic landing [67,214,215] on horizontal surfaces, with the possibility of climbing [216]. Researchers also share developed solutions in the design, construction, and testing of a drone in the form of open source [94], describe the process of designing a drone [217], or develop a drone network [218,219]. In articles, there are also proposals for human–drone interaction using gestures [162], drone communication systems [220], or MNIST character recognition and visual odometry [221].

The use of the mentioned technologies in drones is possible thanks to the use of the following:

- Deep learning [214];
- Decentralised learning [220];
- Reinforcement learning [202];
- Imitation learning [197];
- Simulation [188,189].

### 4.4. Drones as a Source of Risk—16 Documents

Most publications describing non-military drone applications focus their attention primarily on the benefits of introducing drones into ongoing operations. However, many researchers emphasise that drones, while bringing numerous benefits and new opportunities in many fields, can also pose various risks [223]. One of the most common risks is the risk of drone–human collisions [224] or drone-other-machine collisions [225]. In particular, moving amateur drones can lead to serious safety hazards, risks to human life or health, and damage to infrastructure [226].

Publications on potential collisions are characterised in Table 7

Because drones are used to monitor animals and birds, much research has been done on how these creatures react to drones and how these devices may affect certain animal species. Thus, the papers analysed identify publications in which drones are a source of risk to the animals and birds under study:

- The study of how seagulls react to drones [233];
- The study of how 16 bird species react to drone interference in their environment [234];
- The study of the behavioural responses to drones of bison and horses [235];
- The impact of drones on birds [236].

Drones can be a source of noise and visual pollution and are also the subject of ongoing research [237]. In addition, their high vulnerability to weather conditions is also a subject of assessed risk. An example is the research of [29], which analyses the effect of the type of nozzle used (nozzles) on the wind drift rate of a drone when spraying fields.

### 4.5. Cybersecurity—12 Documents

Drones can pose a threat if they are used in an inappropriate or undesirable manner, such as for terrorist attacks. Because they are autonomous devices, the issue of cyber security, therefore, becomes a critical research aspect. Cyberattacks threaten both small, commercial drones used for daily activities [242,243] and drone networks [239,240,241]. Indeed, vulnerabilities in security systems can be exploited by third parties to launch an attack [244]. Cyberattacks can also include GPS forgery, which can be prevented by blockchain technology [245].

Because of the issue’s importance, many researchers are working on systems to protect drones from cyberattacks and data leakage. Such solutions include:

- The privacy-protecting scheme [246];
- The intrusion detection system that monitors network traffic and detects any suspicious or malicious activity [247,248];
- The Internet of Drones identity authentication protocol that provides forward and backward security and resists impersonation attacks [249].

Jahan et al. also proposed an interesting scheme for modelling attacks on autonomous systems. It can be used to analyse the strategy used in drone attacks, which will help better protect drones from potential cyberattacks [250].

### 4.6. Preventive Activities against the Risks Associated with Drones—32 Documents

Because drones are increasingly likely to be a source of occurring risks, a group of publications has also been identified on implementing solutions to mitigate various risks associated with UAV systems. An important aspect of risk mitigation is the early detection of a drone. Detecting drones is becoming increasingly important in the context of public safety, especially concerning the potential risks associated with their illegal use. Detecting drones in surveillance footage is challenging due to their small size, low contrast, and bird similarity. To solve this problem, researchers propose using the following drone detection techniques:

- Deep machine learning [251,252];
- Deep convolutional neural network (DC-CNN) (DC-CNN) [253];
- Spatiotemporal information and optical flow [254];
- Radio frequency (RF) [255,256,257];
- Sensors that measure the sound emitted by the UAV [258];
- The transformer network [259];
- The “fisheye” camera system [260].

Due to the potential collisions in shared airspace, there is a lot of research on increasing air traffic control and managing this traffic in terms of integrating UAVs into urban airspace [261,262,263].

To minimise risks, many researchers are working not only on drone detection systems but also on other types of countermeasures, solutions, and systems to increase the safety of their operations [41,264,265]. These include, for example:

- Solutions to detect the harmful status of a drone [266];
- Risk analysis models [267];
- Systems for collision avoidance [268,269,270,271,272,273,274];
- Systems to prevent UAVs from entering controlled airspace, such as power plants, airports, and military facilities [275].

The European framework defines a set of services and procedures developed as a management system that will enable the organisation of UAS operations and provide users with safe and efficient access to airspace [281].

Procedures for analysing the causes of accidents are also of particular importance. For this reason, Silalahi et al. [276] proposed using log message data to discover and extract some incident-related information using a deep learning-based NLP technique to analyse drone incidents.

The skills of drone controllers are also becoming a critical issue. An essential aspect in this case is the provision of appropriate training equipment that prepares users for the rational and safe use of unmanned aircraft. Examples of such equipment include a device that allows drone control in a virtual environment [277] and a system for inspecting and evaluating drone pilot trainees [278].

Because the source of risk may be not only the drone itself but also its operator, some researchers are conducting studies aimed at investigating who commercial drone users are and what their characteristics entail [279]. Also noteworthy is research into the behaviour of drone users and the methods and techniques they use to reduce the risks involved in drone use, as well as their compliance with airspace requirements and their ability to read visual navigation charts (VNCs) and use AirShare (a local tool that shows airspace requirements) [280].

### 4.7. Opinion Survey—11 Documents

Equipping UAVs with the latest and greatest technology will prove to be a fruitless exercise if potential drone users do not decide to use them. There are doubts in society about the use of drones, so it is important to follow ethical principles when designing drones [284]. User preferences regarding the design and possibilities of using drones were surveyed, and people indicated that drone technology could be used in many outdoor, military, or special missions [285].

It was also checked how people view the following:

- The use of drones as a delivery service [165,167,168,282,283] in medicine [171];
- The use of drones to conduct rescue operations on the beach [286];
- Possible changes in the formation of behavioural intentions of potential users [282];
- Factors that influence a change in the perception of drones [167,168].

Drone users are not only people who choose this parcel delivery method, but also health care workers, and their doubts and opinions must be considered when designing a UAV system for medical use [160,170].

### 4.8. Other—7 Documents

The analyses made it impossible to classify the six documents into any highlighted thematic groups. For this reason, they have been collected in an additional category under the heading “Other”. This includes publications on the following issues:

- Adapting drones for unrestricted and safe indoor use [287];
- The safety of racing drones [288];
- The use of ultrasonic sensors in autonomous devices [289];
- The dangers associated with filming VLOS flights, which arise from the need for the pilot to manage variable supervision at two levels, i.e., filming and flying [290];
- Legal and organisational norms and regulations for the operation and use of drones in various areas [291,293];
- Aspects related to the operation and use of drones [292].

## 5. Discussion

The development of Industry 4.0 and the progressive development of technological UAV systems make it possible to observe a significant increase in the scope of drone applications in non-military areas. This is confirmed both by the growing number of publications on the operation of UAV systems and by the growing number of literature studies related to this research. The results presented in this article represent only a fragment of this research area, which has been consciously limited to issues related to the risks of drone use.

### 5.1. Analysis of the Obtained Results

The analysis of 257 publications enabled the identification and characterisation of eight basic research trends concerning contemporary research in drone application risks. The results of the literature studies indicate that the largest group comprises publications on the application of drones in monitoring certain phenomena or objects, as well as in the processes of transport service and rapid response organisations. This is also confirmed by the data presented in Table 1, according to which the largest group of published literature reviews focuses precisely on the application of drone use in various areas. What should be noted is that none of the drone application publications analysed featured studies on the risks of drone use in military areas. This is, in all likelihood, because most of the results of such studies are confidential due to their critical importance to the military. Therefore, the applied nature of drone risk research focuses primarily on non-military solutions.

The research areas highlighted are not equally numerous. Due to the predominant publications on drone application in this area, two research trends are distinguished:

1. Monitoring (Section 4.1) is the main area of research related to the risks of using UAV systems. Due to the size of this group, we divided it, according to the most popular areas of drone application, into 14 subcategories.
2. Other applications (Section 4.2), where two leading trends can already be distinguished, regarding using drones in transportation and as support in rapid response organisations. This group has the potential for further development as the functionality offered by modern UAV systems expands.

The remaining research categories are far less numerous. Nonetheless, the risk of digital attacks (Section 4.5) was separated from the other risk analyses, in which drones are a source of potential adverse events (Section 4.4). This action was deliberate, as the concept concerning cyber security is now an important research trend, which has its own already defined characteristics and is the subject of separate analyses. In the case of UAV systems, research in this area is particularly important due to the often-autonomous nature of the missions carried out by drones and the high risk of their acquisition by third parties. Therefore, the research described in Section 4.6, which aims to develop solutions and methods to improve the safety of drone use, not only by professionals but also by amateurs, becomes critical. Notably, the latter group of users is a potential source of risk associated with improper drone use, which can cause the occurrence of adverse events. Finally, it is also worth noting that the intensive development of technology related to UAV systems raises more and more societal emotions, including negative emotions such as fear, resistance to deployment, and a sense of discomfort. For this reason, when researching the risks of drone use, one cannot ignore the aspects of public opinion polls on issues related to the sharing of public space or drone use itself. Therefore, the last of the research trends highlighted is the group on public opinion polling (Section 4.7).

Referring to the biometric analysis presented is also necessary when discussing the results obtained. First, the intensive increase in papers published after 2020 is noteworthy. This shows the growing importance of this topic in the last three years, which will continue to develop due to, among other things, observed technological progress and the increasing use of drones in non-military areas of human activity (both industrial and research). The second important point is the strong dominance of publications by researchers from the United States. In second place is China, indicating that significant powers are also investing more and more in non-military use of UAV systems. The low share of publications coming from individual European countries may be worrying. However, when the total number of publications from all countries belonging to the European Union is added up, the result improves significantly. The citation rates of the analysed papers are also evidence of the growing importance of drone research topics. The highlighted publications in the citation analysis have achieved high recognition (number of citations above 50) in the last 2–3 years. This indicates not only the high quality of the results presented but also the popularity of the subject matter covered in them.

### 5.2. Identification of the Research Gap

Although the keywords “drone” and “risk” were used to search for publications for the area under study, it should be noted that risk as an object of study appeared in only a handful of documents. In addition, risks appearing in the text of publications did not refer to a drone performing a mission but to issues related to the phenomenon under study. For this reason, all publications were reanalysed to determine the role played by the drone in the research described. In this way, the authors wanted to confirm the initially identified research gap. The results made it possible to conclude that in only 19 publications out of the 257 documents analysed, the UAV system was the subject of ongoing research. In all of the remaining 238 publications, the drone was merely a tool for conducting research. These highlighted 19 papers were primarily concerned with research in the “Cyber Security” and “Drones as a source of risk” groups. These results indicate that there is currently a lack of research focusing on aspects of risk directly related to drone operations.

In addition, in the highlighted group of 19 publications relating to the risks of drone use, only two articles dealt with the risk assessment method itself. The drone risk assessment process, which includes identifying adverse events and analysing the risk of their occurrence, should consider the specific conditions of drone operation, including the environment in which they operate, the scope of the tasks performed, and the functionality set. Research on this topic could not be identified among the documents analysed. Thus, this area represents a significant research gap that needs to be filled due to the critical nature of this issue from the point of view of further technological development and the scope of drone applications.

The research gaps regarding the risks associated with the areas where drones are used are also worth noting. From the documents analysed, few publications have addressed the risks of drone use in internal logistics operations. Meanwhile, published literature reviews (e.g., [7,179]) indicate that this topic is the subject of numerous studies conducted in this area. Risk assessment models for the use of drones in confined spaces, as well as for internal deliveries feeding production lines and beyond, will become increasingly important with, among other things, the observed steady development of the Logistics 4.0 concept, within which drones are one of the critical cyber-physical systems supporting material flows in the supply chain.

## 6. Conclusions

The increasing scope of use and types of equipment of modern drones make the topics related to the risks of their operation significant from the point of view of safety and the potential and quality of research they can facilitate. Therefore, the research topics addressed in this article are critical from the point of view of further development of this technology and its application in military and non-military conditions. The results presented in this article provide interesting research material for both the industry and academia. Industry representatives can learn about the broad possibilities of drone application in monitoring processes, the technological changes taking place in this area, as well as the risks associated with the operation of the UAV system. The solutions described in Section 4.6 will recognise the latest developments in methods, techniques, and tools to mitigate these risks. On the other hand, representatives of the scientific community can get acquainted with current research trends related to the risks of drone use in industry and also in scientific research. The described research results of the last five years, especially the identified research gaps, can inspire them to develop their research areas and explore applied methods and tools. 

The identified research gap in Section 5.2 also provides a starting point for further research for the authors. Therefore, further research directions will focus on developing risk assessment methods that will consider the specific conditions of drone operation and the purpose of the mission. The second important direction of our further research will be analysing the risks associated with using drones in internal logistics, particularly concerning anthropotechnical systems.

The research conducted is comprehensive in nature. A prestigious journal database—Web of Science—which publishes documents that meet the requirements of the JCR list, was used to search for documents. This ensured the high quality of publications accepted for analysis. Of course, the use of only one journal database and the restriction of documents to publications from only three leading publishers can be considered a limitation of the conducted research. However, such measures were taken consciously, and the results obtained were subject to additional verification. First, an additional search was conducted in the Scopus database per the adopted search model. In most cases, the results overlapped with those obtained from the Web of Science database. Additional publications not included in the database were mostly conference proceedings, which are published in publications that do not have IF points. Thus, they do not have the required scientific impact, the inclusion of which is critical to the formulated conclusions of this research. The second restriction of including publications from the three leading publishing houses was also further verified. After the analysis of the articles selected for this study was completed, publications from two more publishers, Springer Nature and Wiley, were also verified. Both publishing houses published about 30 papers related to the subject matter under study during the analysed period and ranked fourth and fifth on the list of publishing houses publishing this subject matter. The subject matter of the publications from these publishing houses was compared with the proposed classification, and it was found that the subject matter covered was in complete agreement. This means that including these publications in this article would not have had a substantive impact on the identified research trends in the proposed classification framework, but it would have only increased the cited article base. None of these papers included publications that addressed the identified research gap satisfactorily. Therefore, the restrictions introduced can be considered reasonable.

## Figures and Tables

**Table 1:** Two main research streams for drone literature studies.

Research Trend	Scope of the Analysed Literature	Publications
Drone application in the selected area	Mining industry	[6]
	Logistics	[7]
	Last-mile delivery	[8,9]
	Smart city	[10]
	Road traffic monitoring system	[11,12]
	Marine animal research	[13]
	Shark science	[14]
	Clinical microbiology and infectious diseases	[15]
	Healthcare	[16,17,18]
	Agriculture	[19]
	Medical products and transportation	[20]
	Architecture, engineering, and construction industry	[21,22]
	Animal behaviour research	[23]
	Disaster management	[24]
Drone operation	Detection	[25,26,27]
	Social acceptance	[28,29]
	Routing and scheduling problem	[30,31,32,33,34]

**Table 2:** Number of publications in primary sources.

Source Title	Number of Documents	%
Drones	52	20%
IEEE Access	20	8%
Sensors	19	7%
Applied Sciences-Basel	15	6%
Remote Sensing	14	5%
Sustainability	13	5%
Others	124	48%

**Table 3:** Number of publications in main research areas.

Research Area	Number of Documents
Engineering	107
Remote Sensing	69
Computer Science	47
Environmental Sciences and Ecology	44
Chemistry	35
Telecommunications	30
Instruments and Instrumentation	24
Physics	22
Science and Technology—Other Topics	22
Geology	17
Imaging Science and Photographic Technology	17
Materials Science	16
Robotics	10

**Table 4:** The most frequently cited publications.

Authors, Document Title	Times Cited
**Outay, F; Mengash, HA; Adnan, M**	**116**
Applications of unmanned aerial vehicle (UAV) in road safety, traffic, and highway infrastructure management: Recent advances and challenges [38]	
**Shen, Y; Guo, DJ; Long, F; Mateos, LA; Ding, HZ; Xiu, Z; Hellman, RB; King, A; Chen, SX; Zhang, CK; Tan, H**	**93**
Robots under COVID-19 pandemic: A comprehensive survey [39]	
**Shamsoshoara, A; Afghah, F; Razi, A; Zheng, L; Fulé, PZ; Blasch, E**	**71**
Aerial imagery pile burn detection using deep learning: The FLAME dataset [40]	
**Wang, J; Liu, YX; Song, HB**	**71**
Counter-unmanned aircraft system(s) (C-UAS): State-of-the-art, challenges, and future trends [41]	
**Shayea, I; Ergen, M; Azmi, MH; Çolak, SA; Nordin, R; Daradkeh, YI**	**70**
Key challenges, drivers and solutions for mobility management in 5G networks: A survey [42]	
**Annis, A; Nardi, F; Petroselli, A; Apollonio, C; Arcangeletti, E; Tauro, F; Belli, C; Bianconi, R; Grimaldi, S**	**64**
UAV-DEMs for small-scale flood hazard mapping [43]	
**Tarolli, P; Straffelini, E**	**59**
Agriculture in hilly and mountainous landscapes: Threats, monitoring and sustainable management [44]	

**Table 5:** Results of the classification of documents according to the adopted thematic groups.

Basic Category	Subcategories	Eligible Publications
Monitoring	Agriculture	[45,46,47,48,49,50,51]
	Natural disasters	[40,43,52,53,54,55,56,57,58,59]
	Landscape	[44,60,61,62,63,64,65,66,67,68,69,70]
	People	[71,72]
	Animals and birds	[73,74,75,76]
	Plants	[77,78,79,80,81,82]
	Insects	[83,84,85]
	Vehicle traffic	[38,86,87,88,89,90,91,92]
	Water environment	[93,94,95,96,97,98,99,100,101,102,103]
	Infrastructure	[104,105,106,107,108]
	Emission of pollution	[109,110,111,112,113,114,115,116]
	Landslides	[117,118,119,120,121,122,123,124,125]
	Inspection	[38,126,127,128,129,130,131,132,133,134,135,136]
	Other	[137,138,139,140,141,142]
Other applications of drones	Rapid response operations	[39,113,143,144,145,146,147,148,149,150,151,152,153,154,155,156,157,158,159,160,161,162,163,164]
	Transport	[143,144,145,146,147,148,149,150,152,153,160,165,166,167,168,169,170,171,172,173,174,175,176]
	Other applications	[42,177,178,179,180,181,182,183,184,185,186,187]
Technology development related to the operation of drones		[67,94,143,152,154,156,162,163,188,189,190,191,192,193,194,195,196,197,198,199,200,201,202,203,204,205,206,207,208,209,210,211,212,213,214,215,216,217,218,219,220,221,222]
Drones as a source of risk		[223,224,225,226,227,228,229,230,231,232,233,234,235,236,237,238]
Cybersecurity		[239,240,241,242,243,244,245,246,247,248,249,250]
Preventive activities against the risks associated with drones		[41,251,252,253,254,255,256,257,258,259,260,261,262,263,264,265,266,267,268,269,270,271,272,273,274,275,276,277,278,279,280,281]
Opinion survey		[160,165,167,168,170,171,282,283,284,285,286]
Other		[287,288,289,290,291,292,293]

**Table 6:** Characteristics of research areas in publications classified under the “Monitoring” group.

Research Subcategory	Research Area	Publications
Agriculture	Monitoring farmland for decision-making purposes	Cranberries [45] Grass seed [46] Wheat [47]
	Monitoring meteorological variables (temperature, humidity)	Identification of irrigation needs [48] Variables affecting the nutritional value of rice [49]
	Assessment of field damage caused by animals	[43]
	Increasing productivity and food safety	[51]
Natural disasters	Identifying areas at risk and monitoring the occurrence and spread of flood	[43,52,53,54,55,56]
	Identifying areas at risk and monitoring the occurrence and spread of fire	[51,52,53]
	Monitoring the generation of destructive primary lahars responsible for the volcanic eruption	[59]
Landscape	Monitoring the factors responsible for coastal changes	[62]
	Morphological and topographical changes in coastal areas	[60,61,70]
	Monitoring of permafrost	[63]
	Monitoring and 3D modelling of endangered areas	[64,65]
	Gathering spatial information about the landscape	[66]
	Distribution of magnetic minerals in the subsurface	[69]
	Decision support for ecosystem interventions	[44]
	Detection of hazardous obstacles in unstructured terrain environments	[68]
People	Monitoring of sports events	[71]
	Moving people exposed to radiation	[72]
Animals and birds	Detecting bird droppings	[73,74]
	Conducting a census of a selected animal or bird species	[75,76]
Plants	Surveying the vegetation of a selected area	[77,78,79]
	Assessing the state of vegetation destruction in a selected area	[80,81]
	Determination of understory composition	[82]
Insects	Monitoring of insects inhabiting wetlands	[85]
	Identification of breeding sites of virus-spreading insects	[83,84]
Vehicle traffic	Monitoring marine traffic of ships	[92]
	Measurement of speed and behaviour of car drivers, monitoring of traffic volume	[38,86,87,88,89,91]
	Monitoring the driving and dangerous behaviour of cyclists	[90]
Water environment	Sampling and testing of waters	[93,94,95,96]
	Studying the behaviour of sea creatures	[97]
	Ocean observation to model tropical cyclones	[98]
	Monitoring for shark warnings	[99]
	Mapping the distribution of vegetation that provides habitat for sea creatures	[100]
	Monitoring reefs and factors affecting seaweed growth	[101]
	Monitoring critical phenomena to support the management of aquaculture farms	[102]
	Spatial modelling of salt marshes	[103]
Infrastructure	Three-dimensional mapping of selected infrastructure facilities	[106]
	Condition monitoring of transmission networks	[104,105]
	Assessment of the thermal quality of buildings	[107]
	Identification of traces of historical objects	[108]
Emission of pollution	Emission of selected chemical compounds during forest burning	[109]
	Monitoring of air pollution in a selected area	[115,116]
	Measurement of radiological contamination, monitoring of radiation distribution	[110,111]
	Identification of areas where diffuse pollution from agriculture occurs	[112]
	Dispersion of volatile chemicals	[113]
	Mapping of soil contamination	[114]
Landslides	Survey of open pit mine slopes	[122]
	Creating landslide maps for risk assessment	[117,118,119,120,121]
	Monitoring of landslides of rubble and rocks	[123,124]
	Detecting cracks in retaining walls	[125]
Inspection	Conducting inspections of unburied land pipelines	[127]
	Identifying corrosion in industrial structures such as telecommunications towers and wind farms	[129,130]
	Conducting inspections of safety-critical infrastructure	[131]
	Tracking construction progress	[132,133]
	Inspecting terrain for unexploded ordnance and landmines	[134,135]
	Inspecting bridge and road infrastructure	[38,136]
Other	Detection of asbestos roof slates	[137]
	Investigation of orphaned wells	[138]
	Snowpack depth, density, and stratigraphy study	[139]
	Land management	[140]
	Improvement of terrestrial wireless cellular networks	[141]
	Fracture distribution and orientation in outcrops	[142]

**Table 7:** Publications regarding collisions of drones with other objects.

Source of Risk	Characteristics	Publication
Damage to the drone	Engine damage affects flight safety, the surroundings, and the drone’s functionality. There is a possibility of an unplanned impact on the ground.	[227]
Performing missions in a space shared with people and other objects	The possibility of collisions due to changes in the drone’s flight path is caused by difficult weather conditions, in a construction environment where people and machines work among many permanent and temporary structures.	[228]
	Urban areas where the terrain is diverse and there is a high population density.	[229,230]
Performing missions near the airport	Risk of collision with other flying objects.	[225,231,232]

## Data Availability

Data is contained within the article.
